# The complete plastome of *Primula wilsonii*, a heterostylous ornamental species

**DOI:** 10.1080/23802359.2021.1907250

**Published:** 2021-04-08

**Authors:** Yanping Xie, Xingwang Zhang, Xianfeng Jiang, Ganggang Yang

**Affiliations:** a School of Life Sciences, Huaibei Normal University, Huaibei, China; b Hefei Rongxiao Environmental Technology Co., Ltd, Hefei, China; c School of Information, Huaibei Normal University, Huaibei, China; d College of Agriculture and Bioscience, Dali University, Dali, China; e College of Life Sciences, Henan Normal University, Xinxiang, China

**Keywords:** Plastome, *Primula wilsonii*, phylogenetic analysis

## Abstract

*Primula wilsonii* Dunn is a perennial herb in section *Proliferae* Pax of *Primula* L. with small population sizes in the field. Here, we constructed the complete plastome of the *P. wilsonii* using Illumina sequencing technology. The circular plastome was 151,677 bp in size, and comprises a large single-copy (LSC) region of 83,510 bp, a small single-copy (SSC) region of 17,765 bp, and a pair of inverted repeats (IR) of 25,201 bp. The GC content was 36.99% overall, with 34.89%, 30.18%, and 42.87% for the LSC, SSC, and IR regions, respectively. The plastome comprised 130 unique genes including 84 protein-coding genes, 37 tRNAs, and 8 rRNAs. The ML phylogenetic analysis based on 17 plastomes in Primulaceae showed a strong sister relationship with *P. anisodora* in section *Proliferae.*

*Primula* L. is a genus of flowering plants with a heterostylous breeding system and extreme species richness, particularly in the eastern Sino-Himalaya region (Richards 2003). The *Primula* species are of high ornamental value, and are famous garden ornamental flower plants. *P. wilsonii* Dunn is a perennial herb in section *Proliferae* Pax of *Primula* (Primulaceae), which is considered as a well-delimited group characterized by numerous whorls of flowers resembling candelabra (Hu 1990). Unlike the most other candelabra primulas that are common in the wild and gardon, *P. wilsonii* is distributed in Sichuan and Yunnan provinces with small population sizes based on our investigation. Here, we reported plastome of *P. wilsonii* for understanding its systematics and provide scientific basis for formulation of conservation strategy in the future.

Fresh leaves of *P. wilsonii* for total genomic DNA extraction were collected from Wuxuhai, Sichuan Province, China (29.16 N, 101.41E). The voucher specimen (voucher accession number XYP202007016) was stored at the Key Laboratory of Plant Resource and Biology in Huaibei Normal University. The qualified PCR-amplified library was sequenced with the Illumina NovaSeq Tenplatform (Nanjing Genepioneer Biotechnologies Inc., Nanjing, China). The plastome was assembled using the program NOVOPlasty 2.7.2 (Dierckxsens et al. 2016). Annotation was performed using GeSeq (Tillich et al. 2017), followed by manual correction for start and stop codons of protein-coding genes. The assembled complete plastome sequence of *P. wilsonii* was submitted to NCBI, and the accession number is MW442886.

The complete plastome of *P. wilsonii* was 151,677 bp, consisting of a large single-copy (LSC) region of 83,510 bp, a small single-copy (SSC) region of 17,765 bp, and a pair of inverted repeats (IR) of 25,201 bp. The GC content was 36.99% overall, with unevenly distribution across regions of the plastome, which were found to be 34.89%, 30.18%, and 42.87% for the LSC, SSC, and IR regions, respectively. The plastome comprised 130 unique genes including 84 proteincoding genes, 37 tRNAs, and 8 rRNAs. Nine genes (*ndhA*, *ndhB*, *petB*, *petD*, *atpF*, *rpl16*, *rpl2*, *rps16*, *rpoC1*) contained only one intron and two genes (*rps12* and *clpP*) contained two introns. Two protein-coding genes, seven tRNAs, and all 8 rRNAs were completely duplicated within IRs.

To further investigate its phylogenetic position in Primulaceae, especially in section *Proliferae*, a maximum likelihood tree was constructed based on complete plastome sequences of 17 species in Primulaceae by online RAxML BlackBox software (Stamatakis et al. 2008), after the sequences were aligned using MAFFT v7.307 (Katoh and Standley 2013). Our results suggested *P. wilsonii* was close to the other candelabra primulas species, especially closer to *P. miyabeana*, *P. poissonii* and the sister species *P. anisodora* (Figure 1). The cluster including these species are accordant with the results of previous phylogenetic studies of *Primula* (Yan et al. 2011, 2015). This published *P. wilsonii* plastome might provide useful information for phylogenetic and evolutionary studies in section *Proliferae* and Primulaceae.

**Figure 1.**
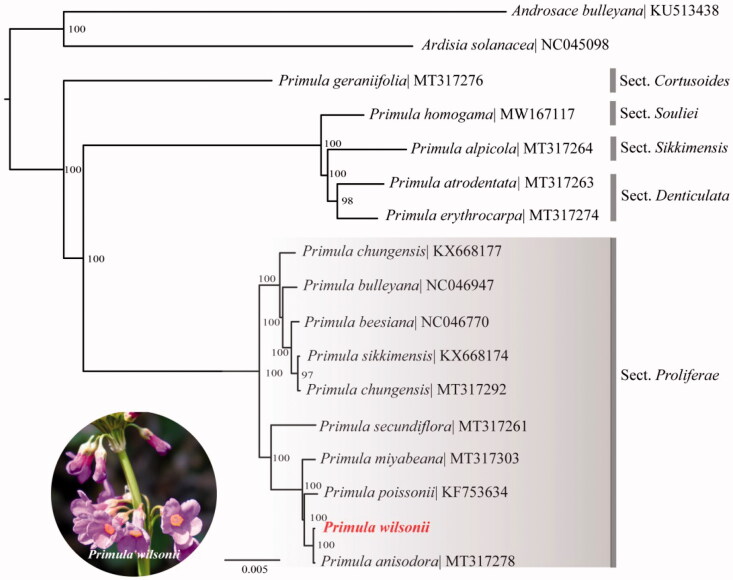
Maximum likelihood tree showing the relationship among *Primula wilsonii* and representative species within Primulaceae. Bootstrap values are indicated for each branch based on 1000 replicates.

## Data Availability

The complete plastome sequence and annotation of *Primula wilsonii* that support the findings of this study are openly available in Zenodo ((doi: 10.5281/zenodo.4435975) at https://zenodo.org/record/4435975#.X_6ybjnivic.
